# Evaluation of childhood cataract characteristics at a tertiary referral center

**DOI:** 10.5935/0004-2749.2021-0303

**Published:** 2023

**Authors:** Ana Paula Silverio Rodrigues, Célia Regina Nakanami, Christiane Regina Rolim de Moura Souza, Nilva Simerem Bueno Moraes, Andréa Araújo Zin, Denise de Freitas

**Affiliations:** 1 Ophthalmology and Visual Sciences Department, Escola Paulista de Medicina, Universidade Federal de São Paulo, São Paulo, SP, Brazil; 2 National Institute of Health of Women, Children and Adolescents Fernandes Figueira, Fundação Oswaldo Cruz, Rio de Janeiro, RJ, Brazil

**Keywords:** Cataract/congenital, Cataract extraction, Diagnostic techniques, ophthalmological, Low vision, Tertiary healthcare, Child, Catarata/ congênito, Extração de catarata, Técnicas de diagnóstico oftalmológico, Baixa visão, Atenção terciária à saúde, Criança

## Abstract

**Purpose:**

To examine the epidemiological characteristics of children undergoing cataract surgery at a referral center in Sao Paulo State, Brazil, as well as the facts surrounding treatment delays.

**Methods:**

In this transversal observational study, 240 operated eyes from 178 children undergoing cataract surgery were reviewed. The following aspects were analyzed: epidemiological and clinical characteristics, parental observations, red reflex test, operated eye, and age at cataract diagnosis and surgery.

**Results:**

The mean ages at the first visit and cataract surgery were 48.9 months (SD=50.0 months) and 64.5 months (SD= 55.4 months), respectively. The most critical sign adverted by parents was leukocoria. The red reflex test was performed on two-thirds of the children, with abnormal results in 28.0%. A family history of cataracts was evident in 30 (20,9%) children (n=144). Previous ocular surgery was found in 37 (16,6%) of the eyes (n=223), anterior segment disorders in 20 (9,0%) eyes (n=221), strabismus in 21 (9,5%) of the eyes (n=220), and nystagmus in 38 (24,4%) of the children (n=156).

**Conclusions:**

One of the causes for the delay in admission may have been the failure to complete the red reflex. In terms of etiology, heredity was the most crucial component. The presence of strabismus and nystagmus in many of these children points to late diagnosis. The most significant impediments to adequate cataract treatment in children were the lack of referral programs and children’s specialized ophthalmologic centers, in addition to the restricted number of support professionals trained in the field and pediatric ophthalmology specialists.

## Abstract (pt)

**Objetivos:**

Rever características epidemiológicas de crianças submetidas a cirurgia de catarata, em centro de referência no estado de São Paulo, Brasil, e fatos associados a atrasos no tratamento.

**Métodos:**

Um total de 240 olhos submetidos a cirurgia de catarata, em 178 crianças, foram revisados neste estudo transversal observacional. Os seguintes aspectos foram analisados: características clínicas e epidemiológicas, sinais apontados pelos pais, teste do reflexo vermelho, olho operado e idade no diagnóstico e na cirurgia.

**Resultados:**

A média de idades na primeira visita e cirurgia de catarata foi de 48.9 meses (DP=50,0 meses) e 64.5 meses (DP=55.4 meses), respectivamente. O sinal mais importante apontado pelos pais foi a leucocoria. O teste do reflexo vermelho foi realizado em dois terços das crianças com resultados anormais em 28%. Histórico familiar de catarata foi evidente em 30 (20,9%) crianças (n=144). Os achados mais prevalentes em termos de histórico de problemas oculares foram: cirurgias oculares prévias em 37 (16,6%) olhos (n= 223), alterações do segmento anterior em 20 (9,0%) olhos (n=221), estrabismo em 21 (9,5%) olhos (n=220) e nistagmo em 38 (24,4%) crianças (n=156).

**Conclusões:**

Uma das causas para o atraso na admissão pode ter sido a falha em realizar o teste do reflexo vermelho, apesar de não ter sido possível verificar se todas as crianças foram submetidas ao exame. A hereditariedade foi o fator mais importante quanto à causa da catarata nessas crianças. A presença de estrabismo e nistagmo mais uma vez aponta para o diagnóstico tardio. Ausência de programas de referência e centros oftalmológicos especializados em crianças, além do número restrito de profissionais de apoio treinados na área e especialistas em oftalmologia pediátrica, foram as barreiras mais importantes para o tratamento adequado da catarata em crianças.

## INTRODUCTION

Childhood cataract is responsible for 5% to 20% of all childhood blindness worldwide^(1)^. It is one of the leading causes of preventable blindness. It leads to a profound loss of vision because these children are not subject to stimulation during sensitive periods of the visual system’s development. This damage may be irreversible^(2)^. Therefore, early diagnosis and treatment are essential for good visual outcomes^(3)^. According to two recent systematic reviews and meta-analyses, the overall prevalence of childhood and congenital cataract ranged from 0.32 to 22.9/1,000 children and 0.63 to 9.74/10,000, respectively. Neither research considered Latin America^(4,5)^. Despite the existing laws on early diagnosis, congenital cataract remains one of the leading causes of visual impairment in low vision services in Brazil^(6)^. The red reflex is a screening test used for the early diagnosis of eye disorders such as cataracts, glaucoma, retinoblastoma, and retinal disorders^(7)^. The present study intends to review the epidemiological characteristics of children referred to university-based hospitals, one of the largest centers in this field in Brazil, as well as the factors associated with delays in care.

## METHODS

In this study, the congenital cataract service was undertaken in the largest and most developed city in Brazil, Sao Paulo. It is one of the largest referral centers of the Unified National Health System. This transversal observational descriptive study was conducted at a tertiary hospital, *Universidade Federal de São Paulo* (UNIFESP), from January 2012 to December 2015. The study protocol was approved by our institutional review board. The subject inclusion criterion included children with lens opacities that interfered with their vision or did not allow fundus examination^(7)^. Epidemiological and clinical characteristics, such as comorbidities, family and ophthalmological history, and ophthalmological findings in the first examination, were analyzed. The signs indicated by parents, red reflex test screening test (RRT), operated eye, and the age of cataract diagnosis, and surgery were also reviewed. We also examined the maternity discharge summaries and childcare documents to analyze whether the RRT had been performed on children of age <12 months. The data collected from the first ophthalmological examination, when available, were also reviewed. The cataract morphology was examined by slit-lamp at the first examination at our service and classified by opacity localization as follows: anterior polar, pyramidal, anterior lenticonus, persistent pupillary membrane, cortices, nuclear, lamellar, sutural and persistent fetal vasculature (PFV), posterior polar, posterior lenticonus, membranous, and total. In the records with incomplete data, the eyes and/or children were excluded in those specific topics where we had a lack of information. This process may be attributed to the different sample sizes (N) in the subsequent results.

The enrolled children were categorized into 4 groups as per their age at the time of surgery, and all cataracts were visually significant, as follows: <12 months (Group A), 13 months to 4 years (Group B), 4-7 years (Group C), and >7 years old (Group D). The data were analyzed descriptively.

The absolute and relative frequencies were applied for categorical variables and summary measures (i.e., mean, quantiles, minimum, maximum, and standard deviation) for numerical variables. The Mann-Whitney U-test was performed to compare the average value of the two groups. A 5% significance level was employed for all statistical tests. One sample binomial test was applied for the comparison of the proportions. For all statistical analyses accomplished, the SPSS 20.0 software was used.

## RESULTS

A total of 178 children who underwent cataract surgery were evaluated, with 56.7% being boys and 43.3% being girls (p=0.084). The mean age at the first visit was 48.9 months (SD=50.0 months). The mean age at the surgery was 64.5 months (SD=55.4 months, median=55 months). The average time between diagnosis and surgery was 15.0 months (SD=19.2 months) (Table1).

**Table 1:** Age at the time of surgery and time from the first examination to surgery in children

	Average	Standard deviation	Minimum	Maximum	1°. Quartile	Median	3°. Quartile	n
Age at surgery (months)	64.5	55.4	1.4	216.4	14.9	55.0	98.6	178
Time from first exam to surgery (months)^2^	15.0	19.2	0.7	101.8	4.3	6.7	15.7	174

Notes: In bilateral cataract surgery, the first eye that had undergone surgery was considered. ^2^ The interval between the date of birth and surgery was considered for toddlers of age <12 months. For children aged >13 months, the first examination date and surgery date were considered.^3^ 5 children had no information on the first examination.

n = total number of children.

We observed similar distribution across the four age groups (p=0.541) (Table 2). in group A, there was no difference in the proportions of unilateral and bilateral cataract proportions (p=0.971). Laterality data was not recorded in groups B, C, and D.

**Table 2:** Age at the time of surgery across the four study groups

	n	%
	**178**	**100.0**
0-12 months (Group A)	38	19.7
1-4 years (Group B)	44	25.3
4-7 years (Group C)	44	24.7
>7 years (Group D)	52	30.3

According to Table 3, no information on RRT was found in more than 1/3 of the children under the age of 12 months (Group A) who had undergone surgery. Among those who had taken the exam, the result was abnormal for 28.0% (95% confidence interval = [12.1%; 49.4%]).

**Table 3:** Red reflex screening of group A (children of age 12 months)

	n	%
**Red reflex screening test**	**38**	**100.0**
No information	11	28.9
Not performed	2	5.3
Performed	25	65.8
**Red reflex screening test result**	**25**	**100.0**
Normal	18	72.0
Abnormal	7	28.0

^1^ Mann-Whitney U-Test to compare the mean values between babies who underwent RRT – p=0.926.

**Graph 1:**
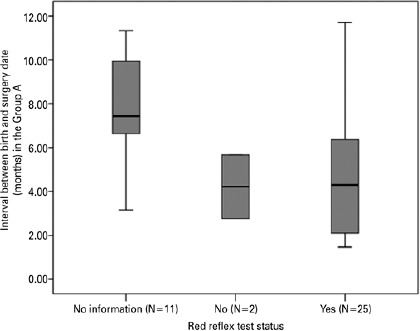
Box-Plot of Interval between the date of birth and surgery (months) in Group A by Red Refex Test Status. Mann–Whitney U-test, p=0.926.

The mean interval between the birth date and the surgery date for Group A was 5.7 months (SD = 3.1 months), and the fact that RRT was performed on children in this particular group did not reduce the interval (4.2 ± 2.1 months *versus* 4.9 ± 3.0) (p=0.926) (Graph 1).

Cataracts, whether unilateral or bilateral, did not affect the detection of an abnormal RRT (p=0.673).

Most patients, 94 (46.3%) eyes (n=203), were referred to our congenital cataract service from other services of the ophthalmology department of UNIFESP. The most common indication reported by parents and caregivers was leukocoria in 54 (26.2%) of the eyes, followed by low visual acuity in 41 (19,9%) of the eyes and strabismus in 16 (7,8%) of the eyes (n=206). Familial history of congenital cataracts was found in 30 (20.9%) children (n=114). The most prevalent comorbidities were prematurity in 20 (12.0%) of the children (n=166), Down syndrome in 15 (9.0%) of the children (n=166), and infection during pregnancy in 2 (1.2%) of the children (n=165). The most common findings in terms of the history of ocular issues were previous ocular surgery in 37 (16.6%) eyes (n=223), strabismus in 21 (9.5%) eyes (n=220), anterior segment abnormalities (for example, persistent fetal vasculature, aniridia, and colobomas) in 20 (9.0%) eyes (n=221), eye trauma in 17 (7.7%) eyes (n=221), cataract following retinoblastoma in 6 (2.7%) eyes (n=221), and glaucoma in 6 (2.7%) eyes (n=221). Nystagmus, esotropia, and exotropia were found in the first ocular examination for 38 (24.4%), 32 (20.4%), and 26 (16.7%) patients of 157 children, respectively. Cataract morphology was classified as total in 62 (28.6%) of the eyes (n=217), lamellar in 48 (22.3%) of the eyes (n=215), nuclear in 35 (16.3%) of the eyes (n=215), and posterior subcapsular in 34 (15.7%) of the eyes (n=216).

## DISCUSSION

A pediatric cataract is a leading cause of preventable childhood blindness in many countries. The etiology varies in different parts of the world and is strongly influenced by socioeconomic factors^(8)^. A blind child’s burden has a significant familial and social impact throughout their lives^(9)^. We had similar sex distribution in our study-101 males (56.7%) and 77 females (43.3%)-which was also found in the literature^(10)^. However, Katibeh et al. revealed that gender differences in the children who were operated on benefited boys^(11)^. Similar results were found in other developing countries where girls had less access to the public health system^(12)^. The average age of the first visit to our pediatric cataract sector was 48.9 months (about 4 years), with a minimum age of 0.4 months (12 days) and a maximum of 199.7 months (16.64 years). Most of the data in the literature consider the age at the surgery rather than the age at the first visit. This study considered the age at the first visit and at the surgery to analyze the duration between the first visit and surgery in our public health system. We found that the average age at surgery was 64.5 months, with 15.0 months delay between the first visit and surgery. Group A (<12 months) shows an improvement between the first visit and surgery, probably due to an enhancement in the application of the RRT screening and, as a consequence, they were referred to tertiary centers sooner. There was no statistically significant difference in the number of surgeries according to age group (Group A, 0-12 months (19.7%); Group B, 1-4 years (25.3%); Group C, 4-7 years (24.7%); and Group D, more than 7 years (30.3%). According to Gogate et al., 14% of the children who underwent bilateral congenital cataracts were aged 0-2 years, 18% were between 3 and 5 years of age, 42.6% were between 6 and 10 years, 20.9% were between 10 and 15 years, and 7.8% were older than 16 years^(13)^. The study by Rajavi et al., which was conducted in a tertiary center in Iran, showed that the age at surgery was similar to our data of children diagnosed with a cataract in the first year of life (5.41 years)^(14)^. The median age at surgery in Toronto was 55 months, while it was 9.1 years in India^(10,13)^. These studies demonstrate that cataracts are prevalent and corroborate that cataracts in children are diagnosed and treated late across the world^(14)^. The ideal timing for extracting a dense congenital cataract is 6 weeks for unilateral cases and between 8 and 14 weeks for bilateral cases because these are critical periods of visual development^(15,16)^. Of the 178 children who had undergone surgery in our study, 38 (19.7%) belonged to the congenital cataract group (Group A). Congenital cataract (defined as opacity detected within the first six months of life) was responsible for 12.4% of surgeries among children in the Gogate et al. research^(13)^. In Group A, there was no predominance of cataract laterality, with 52.6% of the children having bilateral cataracts, and 47.4% had unilateral cataracts, which is consistent with previous research^(4,5,17)^. It was chosen not to record laterality in groups B, C, and D due to a lack of information. Leukocoria (white pupillary reflex) was the most prevalent symptom mentioned by parents in 26.2% of the children who had undergone surgery. More than 10 years ago, in our department, leukocoria was the sign that drew parents’ attention in 80.64% of children who underwent surgery, indicating that parents are probably less aware of this important sign^(18)^. In the study by Haargaard et al., more than two-thirds of the children were examined by an ophthalmologist who was motivated by parents who had noticed a lack of eye contact, strabismus, or a gray/white pupil reflex^(19)^. Mérula and Fernandes, as well as Leite and Zin, pointed out that mothers were usually the first to recognize leukocoria in 38.7% and 62.9% of children, respectively^(20,21)^. Awareness campaigns and parent education are critical allies for early detection^(19,22)^. In different parts of the world, RRT has been regarded as the method of choice for screening ocular diseases during maternity, and it has significant capability to detect earlier congenital cataracts^(18,23)^. After birth, most Brazilian states require this screening test by law. We decided to evaluate RRT exclusively in Group A since we could only verify birth files in children less than 12 months. Our results showed that although most babies underwent the screening test, only 28% had an abnormal result. Fur thermore, the fact that the babies had been submitted to the RRT did not shorten the interval between birth and surgery. The possible causes of such poor results include a lack of proper training and care delays. Rahi and Dezateux concluded that maternity ward scree ning recommendations required intense organizational and educational work^(24)^. Population awareness campaigns for parents are also important allies for early diagnosis^(22)^. For many years, the etiology of congenital cataracts had been attributed to environmental factors, inheritance, and idiopathy^(25)^. Based on the clinical histories of the children who had undergone surgery, we observed that 20.9% had a familial history of childhood cataracts, which was twice as high as that found by Oliveira et al. (9.68%) and Mérula and Fernandes (9.1%)^(18,20)^. This outcome might be explained by a recent shift in behavior regarding the etiology of cataracts in children. Both studies were performed about 10 years before this one ^(18,20)^. Children who had undergone surgery had a history of prematurity (12.0%), Down syndrome (9.0%), metabolic diseases (4.2%), infection (1.2%), and rheumatologic diseases (0.6%). When our study is compared to those done 10 years ago, infectious causes seem to be decreasing at 1.2% against 8.06%, respectively, according to Oliveira et al.^(18)^. Previous eye surgery cases (cataracts at another service, glaucoma, and retina) mostly corresponded to previous ocular issues. Trauma accounted for 7.7% of all the surgeries performed. In the Brandão and Tartarella study, 63.6% of unilateral cataract surgeries were performed due to trauma^(26)^. Cataracts secondary to glaucoma and retinoblastoma surgery also accounted for 2.7% and 2.7% of the surgeries, respectively. Regarding cataract morphology, total cataract was present in 28.6% of the cases, lamellar in 22.3%, nuclear in 16.3%, and subcapsular posterior in 15.7%. These values are similar to those found by Oliveira et al. in the lensectomy of babies undergoing surgery^(18)^. A recently published review showed that the three most common types of cataracts in children are total (31.2%), nuclear (27.2%), and subcapsular posterior (26.8%). It is conspicuous that total cataracts may be the result of an evolution of other cataract forms and are consequently influenced by time^(5)^. Both nystagmus (in bilateral cataracts) and strabismus (mainly in unilateral cataracts) indicate visual impairment. Nystagmus is associated with a change in visual development during the first 3 months of life (critical period). Pre-operative esotropia and exotropia were present in 20.4% and 16.7% of the children operated, respectively, and nystagmus was present in 24.4% of them, according to our study. At the pre-operative ophthalmological exam, nearly half of the children in the Rajavi et al. research showed strabismus^(14)^. These values were reported across all investigations, and early diagnosis and treatment, especially in infants, is essential. Blind children have a greatly familial and social impact due to the years of life they still have to go through^(9,23)^. In recent years, the visual prognosis of children with cataracts has improved. Most pediatric ophthalmologists believe that early diagnosis, proper treatment, correct amblyopia management, advances in microsurgical techniques, and intraocular lenses have all considerably helped the disease’s proper management^(27)^. A good visual result in children with cataracts is dependent on many variables, and it is not an easy task for the ophthalmologist who proposes to treat these patients. In addition to accurate diagnosis and treatment to prevent amblyopia, proper follow-up after surgery is necessary. Frequent ophthalmological exams are essential to update refractive changes and early identification of complications such as visual axis opacification and glaucoma. Occlusive patching, especially for unilateral cataracts, is an amblyopia preventive therapy chosen until the patient no longer poses any risks^(28)^.

In children with cataracts, there is a delay in admission as well as between diagnosis and surgery. The inability to execute the RRT and the fact that the test did not identify an ocular change in 72% of the cases might have been one of the causes of the admission delay.

In this group of operated children, heredity was the most important factor regarding etiology followed by preexisting ocular diseases, genetic syndromes, and infections. The morphological types of cataracts found in this study correspond to the most often reported types in children. The presence of strabismus and nystagmus in many of these children suggests that they were diagnosed late.

The lack of referral programs and children’s specialized ophthalmologic centers, as well as a restricted number of support professionals trained in the field and a low number of pediatric ophthalmology specialists, are likely the most important barriers to adequate cataract treatment in children. In addition to financial investments in pediatric ophthalmologic centers, measures such as spreading awareness, training health care workers, and educating parents, will enhance outcomes and treatment efficacy.
